# EUS-guided FNA of a portal vein thrombus in hepatocellular carcinoma

**DOI:** 10.11604/pamj.2015.21.86.6991

**Published:** 2015-06-03

**Authors:** Yusuf Kayar, Kenan Ahmet Turkdogan, Birol Baysal, Nurcan Unver, Ahmet Danalioglu, Hakan Senturk

**Affiliations:** 1 Bezmialem Vakif University, Department of Internal Medicine, Division of Gastroenterology, Istanbul, Turkey; 2 Adnan Menderes University, Department of Emergency Medicine, Aydin, Turkey; 3 Bezmialem Vakif University, Department of Pathology, Istanbul, Turkey

**Keywords:** Endoscopic ultrasonography, hepatocellular carcinoma, portal vein thrombosis

## Abstract

Portal vein thrombosis is a relatively rare but well-known complication of cirrhosis that has a prevalence of between 1% and 5.7%. On the contrary, in case of hepatocellular carcinoma (HCC), it is a much more frequent complication. In this paper, we presented three cases that had liver cirrhosis, mass and portal vein thrombosis in liver. We were not able to diagnose the cases through imaging methods, laboratory results or histopathologically, however, they were diagnosed with endoscopic ultrasonography- fine needle aspiration EUS-FNA from portal vein thrombus.

## Introduction

Up to 70% of patients with HCC experience tumor invasion into the portal vein (PV) by direct venous extension or metastasis [1]. When patients display a liver mass expressive of HCC and PV thrombus that intensifies on arterial phase of computer tomography (CT) or magnetic resonance imaging (MRI), the diagnosis of a tumor thrombus is relatively easy. On the other hand, when there is no discrete or infiltrating liver mass or when there is nondiagnostic increase of AFP level or equivocal intensification of tumor thrombus, tumor thrombosis diagnosis is difficult. A definite diagnosis or exclusion of tumor thrombus becomes critically important in case of liver transplantation or a curative resection; thus, tissue diagnosis is always confirmatory [2, 3]. The feasibility and safety of fine needle aspiration (FNA) of PV thrombus under transcutaneous ultrasonography guidance has been described by a number of case series. Yet, due to technical difficulties, this is not widely used [1]. However, although there are only few interventions with endoscopic ultrasonography (EUS)-FNA, it has been reported that it should be used as the first choice since it is easy to apply, safe and since it has a high chance of success [2, 4]. In this paper, we presented three cases that had liver cirrhosis, mass and portal vein thrombosis in liver. We were not able to diagnose the cases through imaging methods, laboratory results or histopathologically, however, they were diagnosed with EUS-FNA from PV thrombus.

## Patient and observation

### case 1

48 year-old male patient was hospitalized with a complaint of abdominal pain and nausea that had been going on for about eight months. The patient had no peculiarity in his anamnesis and family history. The patient had been smoking for 10 package/years. His physical examination revealed icteric 2 cm long hepatomegaly and 4 cm splenomegaly. His laboratory results were as follows: Glucose: 86 mg/dl, Creatinine: 0.9 mg/dl, AST: 92 U/L, ALT: 89 U/L, ALP: 139 U/L, GGT: 386 U/L, LDH:162 U/L, Total bilirubin: 6.3 mg/dl, Conjugated bilirubin: 6.0 mg/dl, Albumin:3.2 g/dl, Amylase: 1523 U/L, Lipase: 1254 U/L, Wbc: 5060 mm^3^, Hct: 38.4%, Platelet:109.000 mm^3^ Prothrombrin time: 14 sec, AFP:4.1 ng/mL and HbsAg: positive. In his abdominal ultrasonography (USG) and portal vein doppler USG, the liver had a cirrhotic appearance and splenomegaly was found. His gastroscopy revealed grade III esophageal varicosis. His dynamic contrasted abdominal magnetic resonance (MR) showed intrahepatic bile ducts dillated, choledoch normal, acute pancreatitis and in fourth segment of the left lobe of the liver a mass without typical wash-out but intense contrast in the arterial phase with a size of 10x8 cm and his MR angiography showed thrombus on the portal vein left branch. The result of mass biopsy was reported as focal nodular hyperplasia. Tenofovir was started and his endoscopic ultrasonography (EUS) showed a mass with a diameter of 10 cm and thrombus on the portal vein in the left lobe of the liver. Biopsy was taken from the thrombus with 25 G FNA needle. The biopsy showed big irregular atypical cells in groups some of which were distributed alone and in some places looked like trabecular structures and he was diagnosed as HCC. The patient underwent radiofrequency embolisation and he was taken under control.

### Case 2

50-year-old male patient. The patient had been receiving antiviral treatment for 13 years with a diagnosis of chronic hepatitis B and he was under control. Five years ago, a mass with a diameter of 2 cm was found in the eighth segment of the liver. The patient was operated and his pathology was reported as HCC. The patient who developed abdominal pain in his follow-ups was hospitalized. The patient had no peculiarity in his anamnesis and family history. He had been using tenofovir 245 mg/day and had been smoking for 10 package/years. His physical examination was normal. His laboratory findings were as follows: Glucose: 86 mg/dl, Creatinine: 0.9 mg/dl, AST: 92 U/L, ALT: 89 U/L, ALP: 139 U/L, GGT: 386 U/L, LDH:162 U/L, Total bilirubin: 6.3 mg/dl, Conjugated bilirubin: 6.0 mg/dl, Albumin:3.2 g/dl, Wbc: 5060 mm^3^, Hct: 38.4%, Platelet:109.000 mm^3^, Prothrombrin time: 14 sec, AFP:4.1 ng/mL and HbsAg: positive, HBV DNA: negative. His abdominal tomography (AT) showed a solid mass with a regular contour in the left lobe of the liver next to the lateral segment anterior. The mass had extrahepatic location, it had a size of 80x52 mm, it had a heterogeneous inner structure, it had intense contrast at arterial phase but it did not have typical wash-out. AT angiography showed thrombus at the portal vein left branch. The patient underwent EUS and biopsy was taken from the mass on the left lobe of the liver with 22 G needle and FNA biopsy was taken from the thrombus on the portal vein with 25G needle. The biopsy results of both the mass in the liver and the thrombus were HCC. The patient underwent radiofrequency embolisation and he was taken under control.

### Case 3

77-year-old male patient. The patient was diagnosed with chronic hepatitis B four years ago and started lamivudin 100 mg/day. The patient who did not come to his controls was hospitalized with complaints of swellings on the legs, asthenia and pain in joints. The patient had no peculiarity in his anamnesis and family history and he was not smoking or drinking. His physical examination revealed faintness, moderate edema, 2 cm splenomegaly and arthritis on the left toe and left ankle. His laboratory findings were as follows: Glucose: 86 mg/dl, Creatinine: 0.7 mg/dl, AST: 81 U/L, ALT: 95 U/L, ALP: 56 U/L, GGT: 28 U/L, LDH:217 U/L, Total bilirubin: 0.8 mg/dl, Conjugated bilirubin: 0.5 mg/dl, Albumin:3.3g/dl, Wbc:4550mm^3^, Hct: 30.2%, Platelet:213.000mm^3^, Prothrombrin time: 18 sec, AFP:1.1 ng/mL and HbsAg: positive, HBV DNA: 1.270.000 IU, Anti delta total (+) and HDV RNA (-). His abdominal MR showed cirrhotic liver, a mass lesion with a size of 70x45 mm on the seventh segment of the right lobe. T1A examination showed that the central of the mass lesion was hyperintense, its periphery was hypointense; postcontrasted examination showed that it did not show an obvious contrast but it showed diffusion limits. In the eighth segment of the right lobe of the liver, a centrally located 25x20 mm lesion was seen which extended to the inferior vena cava and caused tumor thrombus in the vena cava. EUS showed masses with diameters of 7 cm and 2 cm on the seventh and eighth segments of the liver and thrombus that extended to the vena cava inferior. Biopsy was taken from the thrombus with 25 G FNA needle. Biopsy result showed HCC. The patient underwent radiofrequency embolisation and he was taken under control.

## Discussion

Although hepatocellular carcinoma (HCC) is a fatal complication of cirrhosis, recent advances in treatment such as liver transplantation in select cases have turned HCC into a potentially curable disease. Attempts of curative treatment options can be made only when extensive vascular invasion or extrahepatic spread are absent. Since the present laboratory tests lack adequate sensitivity or specificity, HCC is usually diagnosed by radiologic imaging [2]. HCC can also be diagnosed through noninvasive radiologic imaging modalities, however, these techniques also do not have adequate accuracy when diagnoses are made without tissue sampling [5]. When a mass is found on imaging, increase alphafetoprotein (AFP) level (>200 ng/mL), or a rising AFP level has a very high positive predictive value in the diagnosis of HCC [2]. Yet, at the time of diagnosis 30% of patients have normal AFP levels and they usually remain low, even with advanced HCC [6]. AFP specificity is close to 100% with such values; however, this causes sensitivity to fall by 45% [7]. The positive predictive value (PPV) of AFP is low, with percentages ranging from 9% to 32% [8]. Although all our cases had tumoral portal vein thrombosis, AFP levels were normal. Conventional imaging studies such as abdominal US, CT and MRI cannot easily distinguish between benign and malignant vein thrombi. On imaging studies, portal vein tumor thrombosis (PVTT) appears as a low density plug within a dilated main or lobar portal vein. With contrast in the arterial phase, this plug enlarges and may have an arterial signal on Doppler ultrasound. Nontumor portal vein thrombosis does not enhance with contrast and does not have any Doppler signal but it has a similar appearance to PVTT [1, 3, 9]. In patients with cirrhosis, PVT may be associated with malignancy, inflammatory, and infectious diseases and hypercoagulable states. Iatrogenic intervention in cirrhosis can also cause PVT. PVT can also be provoked by endoscopic sclerotherapy of esophageal varices and percutaneous ablation or surgery therapies for HCC [1, 3, 9]. Except for malignancy, none of the afore mentioned causes are a contraindication for therapeutic intervention in HCC. In cirrhosis, malignant PVT is a usual complication of HCC and is an indicator of advanced tumoral stage. Thus, in order to make a definitive diagnosis, a fine needle aspiration biopsy is required in many cases. In all our three cases, imaging methods were not typical of HCC and PVTT.

In a great number of previous studies, transabdominal (TA) US-guided FNA has been shown to be effective and safe. In Dusenbery et al's study [10], in biopsies, 39 patients were reported to be positive (81.3%), 3 patients were reported to be dubiously positive (6.2%) and only 6 patients were reported to be negative (12.5%). However, although TAUS-FNA is effective and safe, its use in PVT in order to eliminate malignancy has not become routine. The reasons for this are a great number of tumor induced PVT cases being diagnosed with imaging techniques as a result of the development in CT and MRI, the difficulty of TAUS-FNA process due to obesity and assit in patients with liver cirrhosis, frequent complications such as biliary tract and arteriovenous injury and the distance between the skin and PV [2]. The first case report of EUS-guided FNA of the PV for diagnosis of HCC was published in 2004 by Lai and colleagues [11]. This was followed by a second report in 2007 by Storch et al. [12] EUS-guided FNA of the PV has certain advantages over TA-US. The source of the ultrasound beam can be delivered within 2-3 cm of the PV by an echoendoscope which uses high frequency (10-12 mHz) ultrasound, provides excellent resolution and reliable visualization of the PV, its content, and surrounding tissue and organs. In addition, the FNA needle has to go only a short distance which causes the procedure to be quick and precise [2]. Avoiding the common bile duct and vasculature, particularly collateral vessels, the FNA needle can be positioned directly into the PVT. As a result, complications can be minimized, at least in theory. A lower potential for needle tract seeding is another advantage of EUS-guided FNA over percutaneous US- or CT-guided FNA [4].

## Conclusion

As a conclusion, this study shows once more that EUS-FNA is an easy, safe and effective procedure. We recommend the use of EUS-FNA in order to determine that HCC staging and PVT occurrence due to tumor especially in patients with liver cirrhosis who have been found to have mass and PVT in liver, in patients who cannot be diagnosed as HCC and PVTT with imaging techniques and laboratory results and in patients who undergo local treatment or surgical treatment following a diagnosis of HCC and develop PVT during their follow up.
